# Complete Genome Sequence of a Novel Senecavirus A Isolate from an Asymptomatic Pig in China

**DOI:** 10.1128/MRA.01660-18

**Published:** 2019-04-04

**Authors:** Zhi Zhang, Bo Ni, Lili Zhang, Hui Zhang, Feng Zhang, Shuang Liu, Yaqin Dong, Jin Cui, Faxing Wu, Xiaocheng Li, Baoxu Huang

**Affiliations:** a China Animal Health and Epidemiology Center, People’s Republic of China; University of Arizona

## Abstract

Senecavirus A (SVA) is the single member of the genus Senecavirus of the family Picornaviridae. Here, we report a complete genome sequence of SVA strain GX2018-92, which was isolated from an asymptomatic piglet in China. This virus genome consists of 7,280 nucleotides and will be available to further study the epidemiology of porcine SVA.

## Abstract (precis)

Senecavirus A (SVA) is the single member of the genus Senecavirus of the family Picornaviridae. Here, we report a complete genome sequence of SVA strain GX2018-92, which was isolated from an asymptomatic piglet in China.

## ANNOUNCEMENT

Senecavirus A (SVA) is a single-strand positive-sense RNA virus that belongs to the genus *Senecavirus* of the family *Picornaviridae* (1). The first SVA strain, SVV-001, was identified as a contaminant in the supernatant of the PER.C6 cell line (2). SVA was first found to cause porcine idiopathic vesicular disease (PIVD), in which erosion and vesicles on the pig’s snout and in the oral cavity occur, in Canada (3). Since then, some outbreaks have been extensively reported in the United States, Brazil, China, Thailand, and Columbia (4–8).

Since the first appearance of SVA in China in 2015 (9–11), there were no reports about SVA isolation from pigs without any clinical signs, only from vesicular fluid. Here, we determined a novel complete genome sequence of SVA strain GX2018-92, which was isolated from an asymptomatic pig in a slaughterhouse in China in June 2018. This sample did not show any pathological changes and was verified SVA RNA positive by reverse transcription-PCR (RT-PCR). Its filtered supernatant was then inoculated into BHK cells, and three blind serial passages were carried out until a cytopathic effect was observed. The virus was purified by plaque formation assay, and mRNA was extracted using TRIzol (Thermo Fisher, USA). RT-PCR was carried out using a One-Step PrimeScript RT-PCR kit (TaKaRa Bio, Inc., Japan) together with 8 paired primers (designed according to the sequences of the SVV-001 strain [GenBank accession number NC_011349] and a Chinese strain [GenBank accession number MF893200]) to amplify the whole genome of SVA.

The PCR products were cloned and sequenced (Sangon, China). The 5′- and 3′-terminal sequences were determined by using a kit for rapid amplification of cDNA ends (RACE; 3′ RACE and 5′ RACE). All fragments were amplified and sequenced three times in both directions, and reads were assembled by DNAStar to construct a nearly full-length genome of SVA strain GXT2018-92.

This strain consists of 7,280 nucleotides (nt) and has a structure similar to those of other viruses in the family *Picornaviridae*. It consists of a 668-nt 5′ untranslated region (UTR), a 66-nt 3′ UTR, and an open reading frame (ORF) that maps from positions 659 to 6546 and encodes a 2,182-amino-acid polyprotein. The polyprotein is comprised of a leader protein (L), four structural proteins (VP4, VP3, VP2, and VP1), and seven nonstructural proteins (2A, 2B, 2C, 3A, 3B, 3C, and 3D). Phylogenetic analysis indicated that GXY2018-92 was more closely related to the Chinese strains KY419132 and KY747510 than to any other SVA strains in GenBank. Even though the virus GXT2018-92 was isolated from a subclinical pig specimen, it does not belong to a different evolutionary branch than other strains that can cause vesicular symptoms. The genome sequence of GXY2018-92 shared 97.7% and 93.2% identity at the nucleotide level with those of SVA strains SVA/HLJ/CHA/2016 (GenBank accession number KY419132) and SVV-001 (GenBank accession number NC_011349), respectively. It shares the greatest homology with that of classical strain SVV-001, which indicates that the nucleotide mutations of the epidemic strain were continuing to evolve.

In conclusion, these genome data for GXT2018-92 will facilitate understanding of the molecular evolutionary characteristics of SVA.

### Data availability.

The complete genome sequence of SVA strain GXT2018-92 has been deposited in GenBank under the accession number MK256736.
